# Importance of Mentoring on Workplace Engagement of Emergency Medicine Faculty: A Multi-institutional Study

**DOI:** 10.5811/westjem.2020.11.48510

**Published:** 2021-03-31

**Authors:** Raymond H. Lucas, Valerie Dandar

**Affiliations:** * George Washington University, School of Medicine and Health Sciences, Department of Emergency Medicine, Washington, District of Columbia; † Association of American Medical Colleges, Washington, District of Columbia

## Abstract

**Introduction:**

Mentoring in emergency medicine (EM) has not been well studied despite a larger body of literature that has described the value of mentoring in academic medicine on career satisfaction and scholarly output. Over half of all EM faculty nationally are of junior faculty ranks. The aim of this study was to identify the frequency and types of mentoring in EM, how types of mentoring in EM differ by gender, and how mentoring correlates with workplace satisfaction for EM faculty.

**Methods:**

Using descriptive statistics and chi-squared analysis, we analyzed data from a cohort of medical schools participating in the Association of American Medical Colleges StandPoint Faculty Engagement Survey.

**Results:**

A total of 514 EM faculty from 26 medical schools replied to the survey. Nearly 80% of EM faculty reported receiving some sort of mentoring; 43.4% reported receiving formal mentoring; 35.4% reported receiving only informal mentoring; and 21.2% received no mentoring at all. Women EM faculty received formal mentoring at lower rates than men (36.2% vs 47.5%) even though they were more likely to report that formal mentoring is important to them. Workplace satisfaction was highest for faculty receiving formal mentoring; informally or formally mentored faculty reported higher workplace satisfaction than faculty who are not mentored at all. Unmentored faculty are less likely to stay at their medical school than those formally mentored (69.8 % vs 80.4%).

**Conclusion:**

Institutions and department chairs should focus on mentoring EM faculty, particularly women, to increase engagement and reduce attrition.

## INTRODUCTION

A large body of literature on the importance of mentorship in academic medicine^1^ has demonstrated positive effects on general career satisfaction,^1^–^6^ retention,^1^–^3^,^5^,^6^ and scholarly output.^1^,^2^,^4^,^7^,^8^ Participant (mentee and mentor) satisfaction is the most commonly measured outcome, however. Mentorship is especially important for emergency medicine (EM), given the growth of this newer academic specialty. Prior work by us has shown that compared to other clinical specialties, EM faculty are more likely to be younger and of junior academic ranks, and half of EM faculty have been in their current position five years or less.^9^ There is a much smaller body of literature focused on mentorship in EM^10^,^11^ with few meaningful outcome measures reported. Welch^10^ reported the results of a survey of EM mentoring practices in 2017, but with a low response rate (29%) and responses captured from department chairs only. In our study we sought to expand on previous research to identify the frequency of formal and informal mentorship as reported by EM faculty, how types of mentorship differ by gender, and how the presence of formal mentorship correlates with EM faculty workplace engagement. Our findings may be used by department chairs and other academic leaders to improve the state of mentoring in academic EM.

## METHODS

We used data from the Association of American Medical Colleges (AAMC) StandPoint Faculty Engagement Survey (SFES) from 26 US Liaison Committee on Medical Education (LCME)-accredited medical schools who completed the survey from January 2017–November 2019. The SFES is an optional service offered by the AAMC to help medical schools assess and improve faculty engagement and retention. This validated, web-based assessment was first developed in 2008 by experts in survey design, organizational psychology, and academic medicine,^12^ and since then has been administered to faculty in over 70 US medical schools. Participating institutions have convened annually since 2014 as the StandPoint Faculty Learning Community. This group consists of faculty affairs professionals and representatives from the AAMC who review aggregated survey data and trends and disseminate best practices to improve faculty engagement. We are members of this learning community and thus were granted access to aggregated survey responses for this study.

Most survey questions use five-point Likert scales to assess satisfaction and agreement across 15 dimensions of workplace engagement, including mentoring and feedback. Additionally, the survey measures overall workplace satisfaction and intention to leave one’s job. The StandPoint Survey assessed whether respondents received formal or informal mentoring and whether they received mentoring from within or outside of their institutions. Results from these questions were used to re-code respondents into three distinct groups of individuals for comparison: those who received formal mentoring with or without informal mentoring; those who received only informal mentoring; and those who did not receive either formal or informal mentoring.

We used descriptive statistics and chi-squared analyses to analyze differences between these subgroups of survey respondents using IBM SPSS version 25 (IBM Corporation, Armonk, NY). While chi-square analyses were conducted across the five-point Likert scale responses, percentages presented here reflect the aggregation of the top two response categories for ease of reader interpretation, eg, “very satisfied” and “satisfied,” presented as the percentage of faculty satisfied, across a Likert scale of “very satisfied,” “satisfied,” “neither satisfied nor dissatisfied,” “dissatisfied,” and “very dissatisfied.” We intentionally focused on presenting data as “percent satisfaction,” recognizing that practical workplace interventions are aimed at moving faculty perceptions toward a sense of satisfaction both from those reporting neutral and dissatisfied responses. The American Institutes of Research, the institutional review board of record for the AAMC, approved the StandPoint Surveys data collection and research efforts.

## RESULTS

For the study period, 560 of 860 EM full- and part-time faculty at 26 medical schools responded (65.1%), and 12,251 of 19,938 non-EM clinical faculty responded (61.4%) to the SFES. Of those respondents, 514 EM faculty answered the question about what types of mentoring they received and were included in the analysis. Emergency medicine faculty were more likely to be men (61.8%), identify as White or Asian (90.6%), and hold an assistant professor rank (54.4%). Table 1 summarizes demographic data on the EM survey respondents. To assess generalizability of the SFES sample, this respondent group was compared with the AAMC’s Faculty Roster,^13^ which is a database of all full-time faculty at US LCME-accredited medical schools. Our sample is comparable by gender (61.8% vs 62.4% men) to 2019 reports of full-time faculty in the AAMC Faculty Roster; however, our sample consists of a slightly lower percentage of EM assistant professors (54.4 % vs 58.8%) and racial and ethnicity minority faculty (9.4% vs 12.9%) than nationally reported in the AAMC Faculty Roster. In our study, significantly more EM faculty were assistant professors (54.4% vs 47.5%, *P* = <.001), on non-tenure tracks (80/1% vs 73.8%, *P* =.003), and age 45 or younger (52.5% vs 33.4%, *P* =<.001), compared to faculty in other clinical departments.

**Population Health Research Capsule**

What do we already know about this issue?

*Mentoring improves career satisfaction, retention, and scholarly output. The importance of mentoring specifically in emergency medicine (EM) has not been well studied.*

What was the research question?

*What are the frequency and types of mentoring reported by EM faculty? How does mentoring correlate with workplace engagement?*

What was the major finding of the study?

*Mentorship improves workplace engagement and retention for all EM faculty; women receive less formal mentoring than men.*

How does this improve population health?

*Engaged faculty are important to the population health and advocacy missions of EM. Mentoring is an effective way to improve faculty engagement and retention.*

Table 2 describes the type and frequency of mentoring reported by EM survey respondents and faculty from other clinical departments. Emergency medicine faculty reported receiving more mentoring overall, both formal and informal, than faculty in other clinical departments (78.8% vs 71.3%, *P* = <.001), particularly more formal mentoring (43.4% vs 36.2%, *P* = < .001). For EM faculty, most formal mentoring occurred through their department or medical school, yet 28.6% (n = 64/223) of faculty with formal mentoring reported receiving it through a society or professional organization. Thirty-five percent of EM faculty reported only receiving informal mentoring, and 21.2% reported receiving no mentoring at all.

Table 3 displays the types of mentoring received by EM faculty by gender, race, age, and academic rank. Overall, EM men and women faculty received some type of mentoring at similar rates. However, men received more formal mentoring than women (47.5% vs 36.2%, *P* = .022). Faculty from race and ethnic groups under-represented in medicine (URM) reported higher rates of mentoring than non-URM faculty; however, this was not statistically significant given the low percentage (<10%) of URM faculty in the survey sample (*P* = .268). The percentage of faculty reporting only informal mentoring was similar, approximately 35% across all academic ranks; however, junior faculty were more likely to report formal mentoring and full professors were more likely to report no mentoring. Results by age are similar to those by rank.

Table 4 lists responses regarding perceptions of the importance of mentoring, satisfaction with professional development and advancement, and several components of workplace engagement segregated by mentoring status. Across all survey items, EM faculty with formal mentoring reported higher levels of satisfaction and engagement than EM faculty who received only informal mentoring or no mentoring at all. For faculty who reported no mentoring, over half also reported that mentoring was important to them. The perceptions of the importance of mentoring differed by gender and rank. For example, of those without a formal mentor, more EM women than men agreed that having a formal mentor was important to them (74.8% vs 50.3%, *P* = <.001) (data not shown). With the exception of full professors without mentors, over half of faculty at all other ranks without mentoring reported that formal mentorship was important to them (Table 4).

When examining satisfaction with advancement and opportunities for development, assistant professors with formal mentors were more satisfied with opportunities for professional development than unmentored assistant professors (78.2% vs 29.6%), and were more satisfied with the pace of professional advancement (68.9% vs 27.8%). Similar trends of gaps in satisfaction were observed among associate professors and instructors who did not receive any mentorship.

In looking at measures of overall satisfaction and engagement in the workplace, we found that 86.0% of all EM faculty respondents with a formal mentor, 70.6% of those with only an informal mentor, and 56.2% of those with no mentor were satisfied with their department as a place to work. Across EM faculty of all ranks, those without mentoring reported that they were less likely to stay at their current medical school in the next 1–2 years compared to faculty who received formal mentoring (69.8% vs 80.4%, respectively). For associate and assistant professors, those without formal mentoring reported they were approximately 10% less likely to remain at their institutions. Lastly, those EM faculty without a mentor who agreed formal mentoring was important reported even lower overall satisfaction across survey items and even lower intent to remain at their institution (59.6%) (data not shown) (Table 4).

## DISCUSSION

In our study, nearly 80% of EM faculty received either formal or informal mentoring, which is encouraging given the larger proportion of younger and more junior faculty in EM compared to other clinical disciplines. Also encouraging is the increasing rate of formal mentoring in EM (43%) compared to 33% reported by Mylona^14^ from a prior cohort of SFES responders from 2011–2016. The rate of formal mentoring for all faculty ranks in our study is consistent with a recent report^10^ that 43.6% of academic EM departments sponsored formal mentoring programs. Nevertheless, there appears to remain room for improvement in the amount of formal mentorship provided, particularly to junior faculty who made up over 60% of our study respondents yet reported a rate of formal mentoring of approximately 45%.

Gender disparity in mentorship is well documented in the academic literature,^2^,^10^,^15^ and our study suggests it continues to exist in academic EM. We found that although women EM faculty valued formal mentoring more than men, they received it less. A 2012 study by Welch and colleagues^16^ describes one approach to mentoring women in academic EM using both vertical and facilitated peer mentoring. A prospective method of tracking program outcomes was not described, but participants found the program valuable with an increase in networking opportunities and an improved gender climate in their department. Based on our findings, academic EM leaders should focus on providing additional mentoring opportunities, especially formal programs, for women faculty both within the department and their medical school. When providing mentoring for women faculty, availability and being from the same department or institution may be the most important characteristics women mentees desire of mentors^15^,^17^ and same-gender mentors may be more desirable to URM women faculty than non-URM women faculty^15^,^17^

It is reasonable to focus mentoring efforts on junior faculty; however, mentorship remains important throughout one’s academic career.^18^ Associate professors, who are at risk for plateaus in academic success and delays to promotion to professor, also need continued mentorship.^19^–^21^ In our study, we found that mentoring rates for associate and assistant professors were similar. However, 62% of associate professors with no mentors agreed that having a formal mentor was important to them, higher than unmentored faculty at any other rank. Nearly 80% of professors of EM in our study reported receiving some sort of mentorship. There is a dearth of literature on the mentoring and faculty development needs of senior faculty in EM and other fields. Based on a recent survey of senior faculty,^22^ preparation for retirement and opportunities to mentor others may be important to this group

In our study 29% of EM faculty who received formal mentoring reported they received it through a society or professional organization. While an internal mentor may provide valuable institutional context in the mentoring relationship, external mentors may provide outside perspectives and serve as an important component of a mentoring network. Many of the specialty societies in EM have mentoring programs including the Academy for Women in Emergency Medicine, the American Association for Women Emergency Physicians, the Young Physicians Section of the American Academy of Emergency Medicine, and others. Department chairs and senior faculty should consider referring junior faculty to these programs to augment internal mentoring opportunities.

Our findings suggest that formal mentorship is associated with higher levels of EM faculty engagement compared to informal approaches, yet our study is not an evaluation of any specific mentoring program. In the SFES, informal mentoring is defined as receiving mentorship from a colleague within or outside of one’s institution that is an informal arrangement. In non-academic medicine contexts, informal mentoring appears to improve job success and job satisfaction.^23^ It is unclear whether this holds true in academic medicine or in EM where informal mentoring is much less studied. In one qualitative study of junior pediatric faculty, informal mentoring was acknowledged as a way to develop a “culture of support” but did not fulfill other aspects of successful mentoring.^24^ In combination with formal mentoring, informal mentoring may be an important component to developmental or mentoring networks, which have been shown to be important to success in academic medicine.^25^ Our study suggests that informal mentoring is better than no mentoring at all, but alone may not be sufficient for optimal workplace engagement of EM faculty. More study of the role of informal mentoring is needed.

## LIMITATIONS

As with any survey research, this study may have had selection bias with more satisfied faculty possibly responding at higher rates. Additionally, the survey is designed for faculty in all specialties; therefore, there may be additional factors important to mentoring in EM not captured by this study. The SFES is made available to allopathic AAMC member schools. Generalizability to EM faculty in other settings, such as osteopathic medical schools or community-based academic medical centers not tightly affiliated with a medical school, is not known.

## CONCLUSION

Our study used a validated survey tool from a sample of over 500 EM faculty similar in gender and race to all EM faculty in US allopathic medical schools. We found that 78% of EM faculty at all ranks reported receiving some sort of mentoring, although less than half (43%) had formal mentors. Male faculty received formal mentoring at higher rates that females, even though more women than men agreed that having a formal mentor was important to them. Formal rather than informal mentorship was associated with higher levels of workplace engagement and intention to remain in one’s job. Department chairs and other leaders should evaluate the state of mentoring in their departments, and identify appropriate internal and external mentoring resources for junior and women faculty to optimize faculty engagement and retention.

## Figures and Tables

**Table 1:** StandPoint Survey clinical faculty respondents by demographic categories.

Faculty demographics	Emergency medicine faculty		Other clinical faculty	
	
	560 (n)	100% (%)	12,251 (n)	100% (%)
Full-time	501	89.6	11,037	90.4
Part-time	58	10.4	1,177	9.6
Male	345	61.8	6,820	56.0
Female	213	38.2	5,350	44.0
Non URM (White, Asian)	462	90.6	10,315	89.2
URM (AI, Black, Hispanic/Latino, OPI, Other)	48	9.4	1254	10.8
Full professor	72	13.1	2,703	22.4
Associate professor	135	24.4	2,947	24.4
Assistant professor	301	54.4	5,722	47.5
Instructor or lecturer	45	8.1	686	5.7
Administrative title	262	48.5	5,336	45.0
Non-administrative title	278	51.5	6,513	55.0
Active clinical	499	96.9	9,490	85.2
Not active in clinical care	16	3.1	1,646	14.8
On tenure track/tenured	104	19.9	3,004	26.2
Not on tenure track	419	80.1	8,479	73.8
LGBT	20	5.1	301	3.4
Non-LGBT	372	94.9	8,471	96.6
Age 45 and younger	262	52.5	3,507	33.4
Age 46 and older	237	47.5	7,002	66.6

*EM*, emergency medicine; *AI*, American Indian or Alaska native; *OPI*, native Hawaiian or other Pacific Islander; *URM*, race or ethnicities under-represented in medicine; *LGBT*, lesbian, gay, bisexual, and transgender.

**Table 2:** Faculty mentoring status.

	Emergency medicine faculty N (%)	Other clinical faculty N (%)	Chi square comparing EM with other clinical faculty
Mentoring survey item (Check all that apply)			
I receive formal mentoring through my department or the medical school	201 (38.3%)	3,533 (30.9%)	*P* = <.001
I receive formal mentoring through a society or professional organization	64 (12.2%)	1,373 (12.0%)	*P* = .905
I receive informal mentoring from a colleague at this medical school	270 (51.4%)	5,359 (46.9%)	*P* =.042
I receive informal mentoring from a colleague at another institution	161 (30.7%)	3,010 (26.3%)	*P* = .028
I receive no formal or informal mentoring*	109 (20.8%)	3,211 (28.1%)	*P* = <.001
Combined mentoring variable (unduplicated)			
Receives formal mentoring	223 (43.4%)	4,054 (36.2%)	*P* = <.001
Receives only informal mentoring	182 (35.4%)	3,942 (35.2%)	
Receives neither formal nor informal mentoring	109 (21.2%)	3,211 (28.7%)	

* This survey question allowed respondents to check more than one choice, except for the response “I receive no formal or informal mentoring,” which was an exclusive choice selection.

**Table 3:** Faculty mentoring status by demographics.

	Emergency medicine faculty			Other clnical faculty		
	
	Formal mentoring N (%)	Informal mentoring only N (%)	No mentoring N (%)	Formal mentoring N (%)	Informal mentoring only N (%)	No mentoring N (%)
All faculty	223 (43.4)	182 (35.4)	109 (21.2)	4,054 (36.2)	3,942 (35.2)	3,211 (28.7)
Male	150 (47.5)	99 (31.3)	67 (21.2)	2,335 (36.7)	1,989 (31.3)	2,031 (32.0)
Female	71 (36.2)	83 (42.3)	42 (21.4)	1,704 (35.6)	1,930 (40.3)	1,158 (24.2)
Non-URM	189 (44.0)	151 (35.1)	90 (20.9)	3,442 (35.9)	3,383 (35.3)	2,752 (28.7)
URM	23 (50.0)	18 (39.1)	5 (10.9)	468 (40.2)	408 (35.1)	288 (24.7)
Full professor	24 (34.8)	24 (34.8)	21 (30.4)	693 (27.1)	791 (31.0)	1,071 (41.9)
Associate professor	54 (42.9)	48 (38.1)	24 (19.0)	892 (32.4)	1,065 (38.7)	792 (28.8)
Assistant professor	123 (44.9)	95 (34.7)	56 (20.4)	2,183 (42.2)	1,854 (35.8)	1,141 (22.0)
Instructor or lecturer	18 (46.2)	14 (35.9)	7 (17.9)	230 (40.4)	179 (31.5)	160 (28.1)
45 and younger	123 (49.4)	88 (35.3)	38 (15.3)	1,688 (49.3)	1,251 (36.5)	486 (14.2)
46 and older	87 (37.8)	78 (33.9)	65 (28.3)	2,088 (30.7)	2,317 (34.1)	2,399 (35.3)

*EM*, emergency medicine; *URM*, race/ethnicity is under-represented in medicine.

**Table 4:** Perceptions of opportunities for growth and global engagement by faculty mentoring status and rank.

	Emergency medicine faculty		

	Formal mentoring	Informal mentoring only	No mentoring
% Agree having a formal mentor is important to me			
All EM faculty	78.1	65.7	52.8
Full professor	66.7	45.8	26.3
Associate professor	75.9	65.2	62.5
Assistant professor	81.7	69.9	56.4
Instructor or lecturer	76.5	71.4	57.1
% Agree are satisfied with pace of advancement			
All EM faculty	75.1	52.0	37.1
Full professor	91.7	75.0	78.9
Associate professor	82.7	56.5	33.3
Assistant professor	68.9	44.6	27.8
Instructor or lecturer	66.7	42.9	14.3
% Agree are satisfied with opportunities for professional development			
All EM faculty	77.5	54.0	32.4
Full professor	87.5	66.7	63.2
Associate professor	73.6	47.8	25.0
Assistant professor	78.2	52.7	29.6
Instructor or lecturer	66.7	57.1	0.0
% Satisfaction with department			
All EM faculty	86.0	70.6	56.2
Full professor	82.6	75.0	70.0
Associate professor	84.6	67.4	45.8
Assistant professor	87.3	72.8	59.3
Instructor or lecturer	82.4	64.3	33.3
% Satisfaction with school			
All EM faculty	79.4	62.7	53.3
Full professor	78.3	75.0	70.0
Associate professor	78.8	58.7	54.2
Assistant professor	80.5	63.0	51.9
Instructor or lecturer	76.5	57.1	16.7
% Unlikely to leave school in 1–2 years			
All EM faculty	80.4	69.0	69.8
Full professor	90.5	81.0	86.7
Associate professor	80.4	63.6	69.6
Assistant professor	82.1	71.4	71.2
Instructor or lecturer	68.8	57.1	16.7

*EM*, emergency medicine.
